# Association of knockdown resistance mutations with pyrethroid resistance in *Aedes aegypti*, a major arbovirus vector in Cameroon

**DOI:** 10.1186/s13071-025-06943-4

**Published:** 2025-07-24

**Authors:** Christophe R. Keumeni, Aurelie P. Yougang, Flobert Njiokou, Sian E. Clarke, Jo Lines, Charles Wondji, Basile Kamgang

**Affiliations:** 1 Centre for Research in Infectious Diseases, Yaoundé, Cameroon; 2 Department of Animal Biology and Physiology, Faculty of Science, University of Yaoundé I, Yaoundé, Cameroon; 3 Vector Biology Department Liverpool School of Tropical Medicine, Liverpool, UK; 4 Disease Control Department, London School of Hygiene and Tropical Medicine, London, UK

**Keywords:** *Aedes aegypti*, Arbovirus, Pyrethroid resistance, Clothianidin, *Kdr* mutations, Cameroon

## Abstract

**Background:**

The development of insecticide resistance in *Aedes* mosquitoes has been reported in several African countries. However, information about the mechanisms involved remains scarce. This study aimed to address this issue by updating the resistance profile of *A. aegypti* and evaluating the role of known knockdown resistance (*kdr*) mutations in the observed phenotypic resistance in *Ae. aegypti* in Cameroon.

**Methods:**

Larvae and pupae of *Aedes* were collected in 2022 in four sites in Cameroon and reared to adulthood. Adult mosquitoes were tested using World Health Organization (WHO) tube bioassays for pyrethroids, bendiocarb and fenitrothion, synergist assays with piperonyl butoxide (PBO) and WHO bottle tests for clothianidin following WHO recommendations. Dead and live mosquitoes after exposure to deltamethrin and permethrin insecticides were used for the genotyping of the *F1534C*, *V1016I* and *V410L* mutations, sequencing of fragments of the voltage-gated sodium channel (*VGSC*) gene and assessment their association with observed resistance.

**Results:**

The analyses revealed that *A. aegypti* exhibited high resistance to all of the tested pyrethroids. Mortality rates ranged from 0% for alphacypermethrin 0.05% in Douala to 63.57% for deltamethrin 0.3% in Yaoundé. An increase in resistance was also observed for 0.1% bendiocarb, with mortality rates ranging from 50.54% in Douala to 68.31% in Garoua. Full susceptibility was observed with 1% fenitrothion. Partial or full recovery of mortality was reported following pre-exposure to a synergist. This suggests the involvement of cytochrome P450 genes in the observed resistance, although other mechanisms may also be involved. The F1534C, V1016I and V410L mutations were found in live and dead mosquitoes in Douala, Yaoundé and Bertoua. However, the V1016I and V410L mutations were more prevalent in alive mosquitoes than in dead ones, indicating an association between pyrethroid resistance and these mutations. After a 1 h exposure, clothianidin showed full susceptibility in samples from Bertoua, Douala and Garoua after 7 days of observation. In Yaoundé, probable resistance was observed with a mortality rate of 94.3%.

**Conclusions:**

These findings provide evidence that metabolic and *kdr* resistance are both involved in *A. aegypti* resistance to insecticides in Cameroon. This should be considered when implementing arbovirus vector control strategies and insecticide resistance management in the country.

**Graphical Abstract:**

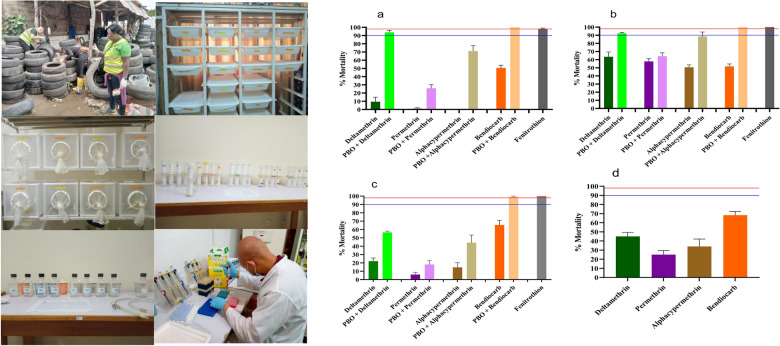

**Supplementary Information:**

The online version contains supplementary material available at 10.1186/s13071-025-06943-4.

## Background

The mosquito *Aedes aegypti* Linnaeus, 1762 (Diptera: Culicidae), also known as the yellow fever vector, is one of the main vectors of several arboviral diseases including dengue, Zika and chikungunya in the tropical and subtropical regions [1, 2]. This mosquito species is mainly active during the day, resting indoors or outdoors and breeding in manmade containers in and around habitations [3–5]. *Aedes aegypti* is present across Cameroon and has been shown to be able to transmit dengue [6], Zika [7] and yellow fever [8] viruses in different domestic environments. In the absence of a specific treatment or an effective vaccine for most of these diseases, vector control remains one of the most effective ways of preventing their transmission [9]. Vector control relies on the destruction of larval habitats and insecticide-based interventions. The use of larvicides such as *Bacillus thuringiensis israelensis* (*Bti*) or temephos to treat water-holding containers, and the space spraying of adulticides in emergencies, can help to reduce the density of *Aedes* mosquitoes [10, 11]. However, many vector control programmes are struggling with the development of resistance to the various classes of insecticide used to control *Aedes* vectors [12–15]. The presence of insecticide resistance in *Aedes* mosquito has been reported in at least 57 countries [16], with higher levels of resistance evident in Asia and South America [15]. Given the evidence of increasing resistance in vectors to the four main classes of insecticides deployed (i.e. organochlorines, pyrethroids, organophosphates and carbamates) [15, 17], new insecticide molecules are being developed. A notable example is clothianidin, a neonicotinoid insecticide, which has demonstrated efficacy in targeting vectors [18], presenting an alternative for vector control measures.

Several mechanisms have been identified to cause insecticide resistance across the world: cuticular [19, 20], behaviour [21], metabolic [22] and target site [23, 24]. Target site resistance, one of the main mechanisms is caused by mutations in target genes such as acetylcholinesterase (*Ace-1*), the *GABA* receptor and the voltage-gated sodium channel (*VGSC*), causing knockdown resistance (*kdr*). One of the most important target site resistance conferring resistance to pyrethroids is *kdr* resistance [25]. More than 12 *kdr* mutations in *VGSC* domains I–IV have been identified in *A. aegypti* around the world [15, 26, 27], and the link between *F1534C*, *V1016G*, *I1011M* and *V410L* mutations and pyrethroid resistance has been established [15, 26, 28].

In Cameroon, previous studies reported that *A. aegypti* populations were resistant to several insecticides such as permethrin, deltamethrin and bendiocarb [29–33]. Similarly, four *kdr* mutations have already been detected in this species in Cameroon, including *F1534C*, *V1016I*, *V1016G* and *V410L* [29, 31, 32, 34]. However, no study to date has established the link between the presence of these *kdr* mutations and the phenotypic resistance observed to insecticides in *Aedes* mosquitoes.

The present study aimed to address this important gap by updating the insecticide resistance profile of *A.* *aegypti* including to clothianidin and investigating the association between these *kdr* mutations and the insecticide resistance observed in *A. aegypti* in different localities in Cameroon.

## Methods

### Mosquito sampling and rearing

**Fig. 1:**
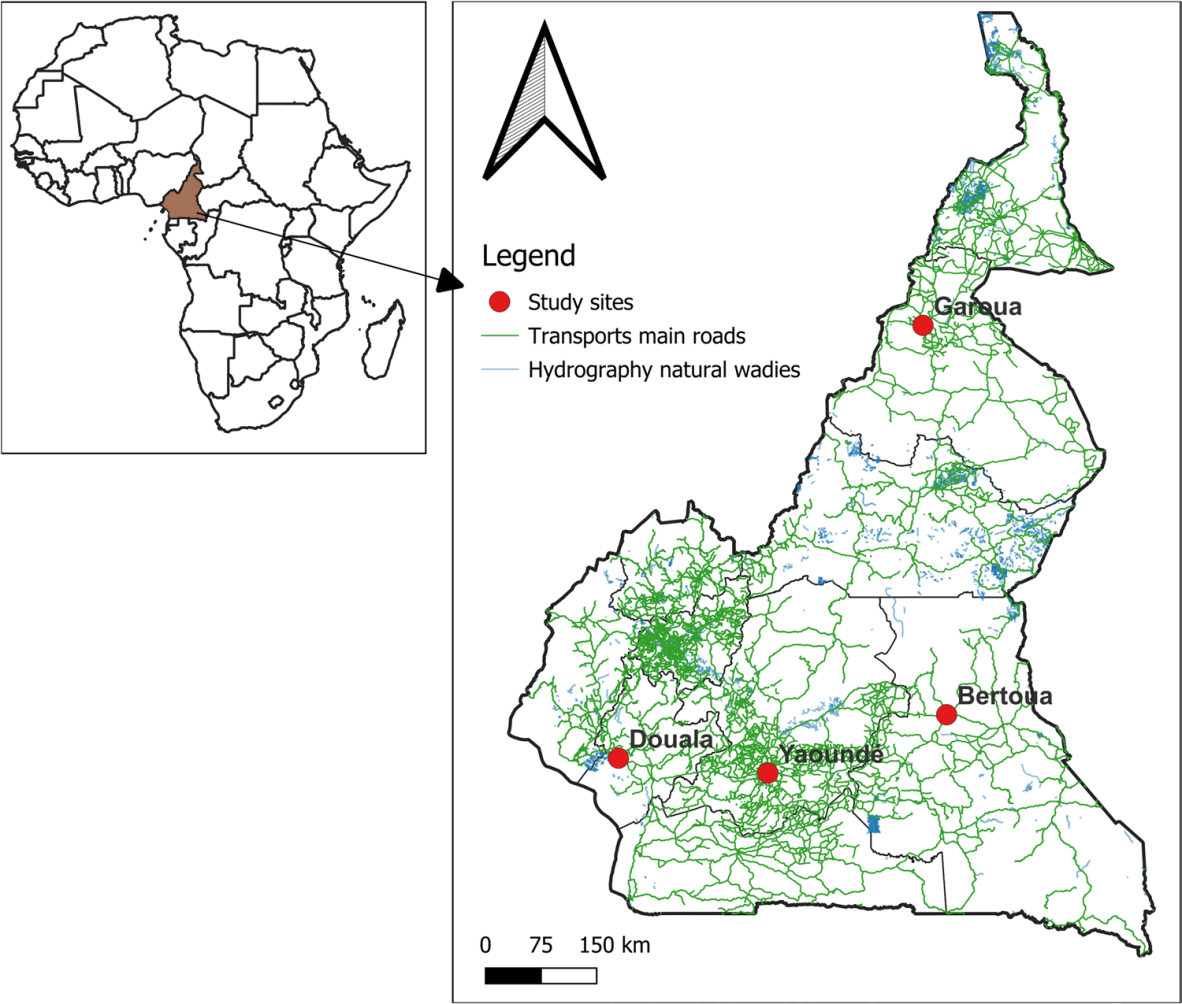
Map of Cameroon showing the locations of study sites. The red dots indicate the study sites; the green lines represent the main transport roads and the blue line shows the natural wadis

Immature stages of *Aedes* mosquitoes were collected during the rainy season between June and October 2022 in four cities across Cameroon: Douala (04°02′53″N, 09°42′15″E), Garoua (09°18′05″N, 13°23′51″E), Yaoundé (03°52′00″N, 11°31′00″E) and Bertoua (04° 34′30″N, 13°41′04″E) (Fig. 1). In each city, larvae or pupae were collected in four neighbourhoods (two in downtowns and two others in suburb) from different larval habitats including used tyres, discarded tanks and car wrecks. In each neighbourhood, larvae or pupae were collected from around 25 larval habitats, stored in plastic boxes, and transported to the insectary, pooled by location, reared to adults (field generation, G0) and morphologically identified [35, 36]. Mosquitoes identified as *A. aegypti* were maintained at the insectary and reared controlled conditions (temperature 27 ± 2 °C; relative humidity 80 ± 10%) until generation G1/G2, for insecticide susceptibility testing. *Aedes aegypti* Benin strain was used as a lab-susceptible strain [37].

### WHO tube bioassay tests

Bioassays was carried out according to WHO protocol [38]. Mosquitoes were tested with the following insecticides: 0.4% permethrin (type I pyrethroid), 0.03% deltamethrin, 0.05% alphacypermethrin (type II pyrethroid), 0.1% bendiocarb (carbamate) and 1% fenitrothion (organophosphate). A total of four replicates of 20–25 unfed, 3–5-day-old female *A. aegypti* were exposed to insecticide-impregnated papers for 1 h under the controlled conditions described above and then transferred to observation tubes and fed with 10% sugar solution. Mortality was recorded after 24 h, and mosquitoes alive or dead were stored in RNA later or silica gel, respectively.

### Synergist assays

Synergist assays were performed to evaluate the involvement of cytochrome P450 genes in metabolic resistance, using 4% piperonyl butoxide (PBO). Adult mosquitoes aged 3–5 days were first exposed to PBO-impregnated papers for 1 h then transferred to a tub containing insecticide for another hour. Mortality was recorded after 24 h and compared with the results obtained using each insecticide without pre-exposure to PBO, in accordance with WHO standards [38].

### WHO bottle bioassay tests with clothianidin

Stock solution was prepared by diluting a technical-grade formulation of clothianidin (PESTANAL^®^, analytical standard, Sigma-Aldrich, Dorset, UK) with acetone and adding a vegetable oil ester, Mero^®^, as a surfactant for a final concentration of 20 μg/mL. The bottles were prepared and biological tests carried out according to WHO protocol [38] using unfed G1 or G2 adult females aged between 2 and 5 days. A total of four replicates of 20–25 mosquitoes per bottle were tested. After the exposure time of 1 h as recommended by the WHO and 30 min to evaluate whether the product remained efficient, the mosquitoes were removed from the bottles and transferred to cups covered with net. Mortality was recorded daily for seven consecutive days.

### Knockdown resistance (*kdr*) genotyping

To investigate if the resistance observed in different samples was due to a target site modification mechanism, we looked for three *kdr* mutations (*F1534C*, *V410L* and *V1016I*) already reported in Cameroon [29, 31, 32, 34] and which have been described as implicated in pyrethroid resistance in *A. aegypti* in other parts of the world [15, 39]. To this end, we prioritised the locations where F1534C mutation was previously detected in Cameroon. Genomic DNA was extracted individually from around 30 dead and 30 live mosquitoes (if the numbers permitted) using Livak’s protocol [40]. These mosquitoes were obtained from insecticide-susceptible tests using permethrin and deltamethrin. Genotyping of the three *kdr* mutations was performed by the melting-curve real-time quantitative polymerase chain reaction (qPCR) using the protocol described by Saavedra-Rodriguez et al. [41, 42]. Each qPCR reaction was performed in a final volume of 21.5 μL containing 10 μl of SYBR^®^ Green, 1.25 μl of each primer, 5.75 µl of sigma water and 2 μl of DNA sample. The Agilent^®^ Technologies Stratagene Mx3000P platform was used for this analysis under the following conditions: 95 °C for 3 min, followed by 40 cycles of (95 °C for 20 s, 60 °C for 1 min and 72 °C for 30 s) then a final step of 72 °C for 5 min.

### Amplification and DNA sequencing of fragments of the voltage-gated sodium channel (*VGSC*) gene in *Aedes aegypti*

To confirm the presence of these *kdr* mutations and assess the polymorphism of the *VGSC* gene, three fragments of this gene covering *V410L* (domain I), *V1016I* (domain II) and *F1534C* (domain III) mutations were separately amplified and sequenced in dead and live mosquitoes from the permethrin exposure. To achieve this, DNA extracted from *A. aegypti* specimens was amplified using specific primers [42, 43]. PCR reactions was performed using 0.51 μL of each primer and 1 μL of genomic DNA as template in 15 μL reactions containing 1.5 μL of Kapa Taq buffer (buffer A), 0.75 μL of MgCl2, 0.12 μL of dNTP, 0.12 μL of Kapa Taq and 10.49 μL of double-distilled water (ddH_2_O). The reaction conditions were as follows: initial denaturation for 3 min at 94 °C, 35 amplification cycles (30 s at 94 °C, 30 s at 66 °C, 30 s at 72 °C) followed by a final elongation for 10 min at 72 °C for domains II and III. After an initial denaturation of 3 min at 94 °C, 35 amplifications cycles were carried out (30 s at 94 °C, 30 s at 57 °C, 45 s at 72 °C) followed by a final elongation of 10 min at 72 °C for the domain I. PCR amplicons were analysed by agarose gel electrophoresis and visualised under UV light. Amplified fragments of the expected size were purified using ExoSAP according to the manufacturer’s recommendations and sent directly for sequencing.

The sequences were manually corrected with BioEdit software (v 7.2.5, London Information Retrieval Ltd, London, UK) and aligned with Clustal W [44]. DNA sequence polymorphism (DnaSP) (v 6.12.03, Universitat de Barcelona, Barcelona, Spain) [45] was used to define the haplotype phase and compute the genetic parameters including the number of haplotypes (*h*), the number of polymorphism sites (S), haplotype diversity (Hd) and nucleotide diversity (*π*). Demographic stability was estimated using statistical tests of Tajima [46] and Fu Fs [47] with DnaSP. Different haplotype sequences obtained and reference sequences downloaded from GenBak were used to construct the maximum likelihood phylogenetic tree using Mega 11.0.13 [48]. A haplotype network was then constructed using TCS [49] and TcsBu [50] programs to further evaluate the genealogical relationship between haplotypes.

### Data analysis

The susceptibility of *A. aegypti* to insecticides in the WHO tube bioassay, WHO bottle assays tests and synergists tests was interpreted following WHO guidelines [38]. A susceptible strain is indicated by mortality rates of mosquitoes between 98% and 100%, a probable resistance strain by rates between 90% and 97% and a confirmed resistance strain by rates below 90%.

For synergist assays, the effect of PBO cannot be reliably assessed if the mean mortality in the ‘insecticide only’ samples is ≥ 90%. However, if the mean mortality in the ‘insecticide only’ samples is < 90%, the effect of PBO can be interpreted according to the following criteria: (1) mean mortality of at least 98% indicates a full recovery of susceptibility to the insecticide after pre-exposure to PBO, suggesting that a monooxygenase-based resistance mechanism fully accounts for the expression of the resistant phenotype in the tested population; (2) partial restoration of susceptibility after pre-exposure to PBO implies that a monooxygenase-based resistance mechanism only partially accounts for the expression of the resistant phenotype, and that other resistance mechanisms are likely to be present in the test population and (3) no restoration of susceptibility after pre-exposure to PBO implies that the detected resistance phenotype is not based on monooxygenase-mediated detoxification. A chi-squared test was performed to evaluate whether the difference in mortality rates with and without pre-exposure to PBO was significant.

Fisher’s exact test was computed for association between genotype and the resistance phenotype using GraphPad Prism 8.0.2 software (GraphPad Software, San Diego, California, USA). A test was considered as statistically significant if the *P*-value was less than 0.05.

## Results

### Adult bioassays

Bioassays carried out with the Benin lab susceptible strain *A. aegypti* confirmed that this strain was susceptible to all the insecticides tested. The mortality rate in controls non-exposed to insecticides was less than 5%.

### Insecticide resistance profile for *Aedes aegypti*

**Fig. 2:**
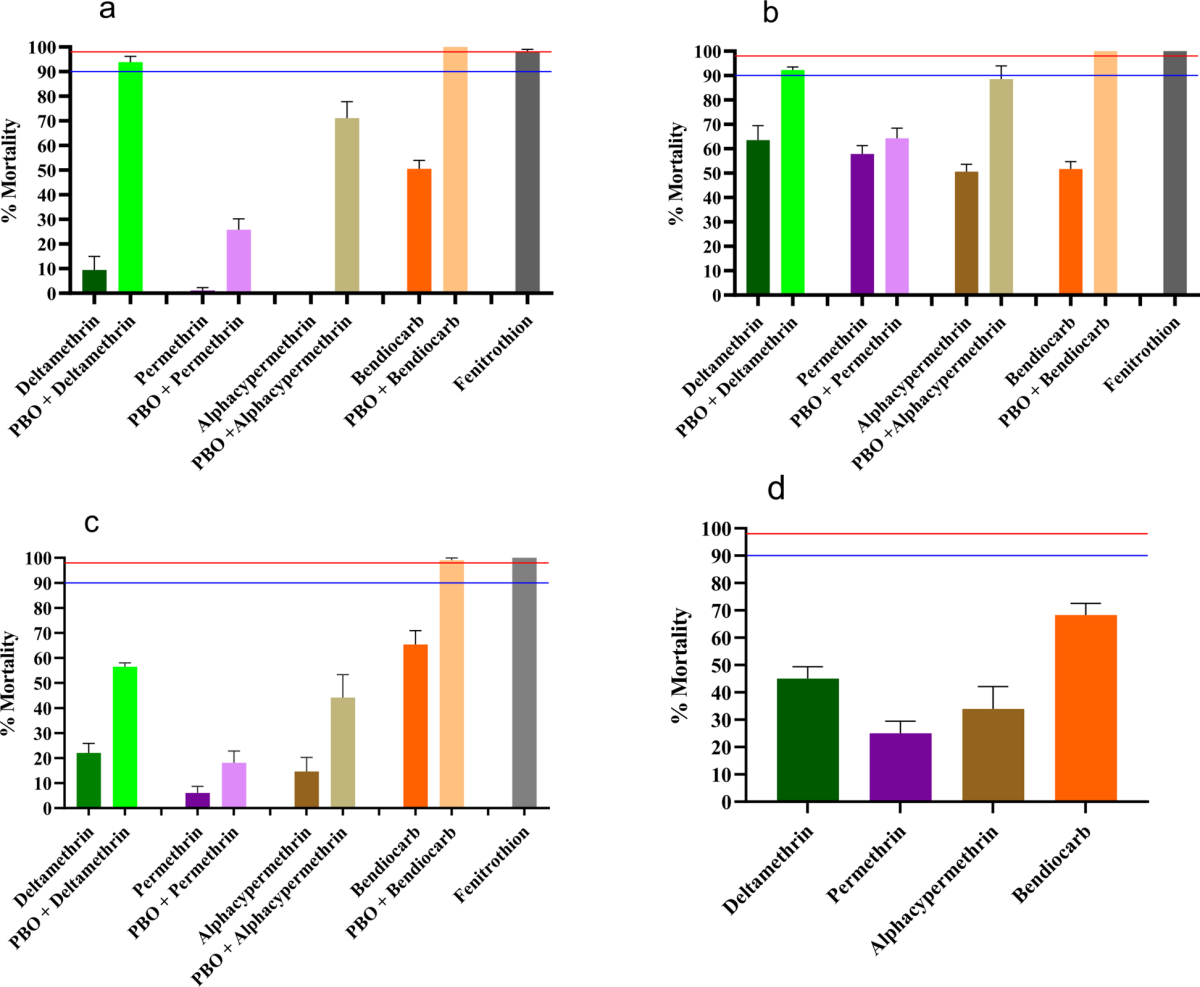
Mortality rate of *Aedes aegypti* 24 h after 1-h exposure to insecticides alone or with 1-h pre-exposure to the synergist PBO. PBO, Piperonyl butoxide; **a**–**d**, mortality rates in Douala, Yaoundé, Bertoua and Garoua, respectively; blue line, probable resistance threshold; red line, susceptibility threshold; error bars represent standard deviation

A total of four populations were tested with all five insecticides, except for the Garoua population, which was not exposed to fenitrothion (Fig. 2). All samples tested were fully susceptible to fenitrothion but were resistant to the other insecticides tested. A high level of resistance was found to 0.40% permethrin with mortality rates ranging from 1.13% in Douala to 57.88% in Yaoundé, to 0.3% deltamethrin with mortality rates ranging from 9.44% in Douala to 63.57% in Yaoundé and to 0.05% alphacypermethrin with mortality rates ranging from 0% in Douala to 50.65% in Yaoundé. Increased resistance was also observed to 0.1% bendiocarb, with mortality rates ranging from 50.54% in Douala to 68.31% in Garoua. To note, the lowest mortality rates to all insecticides were recorded in Douala. This observation indicates that the population from Douala was the most resistant of all those tested.

### Synergist assay with PBO

After pre-exposure to PBO, *A. aegypti* populations showed a partial or full recovery of susceptibility to permethrin, deltamethrin, alphacypermethrin and bendiocarb (Fig. 2).

Partial restoration of susceptibility was observed to permethrin in the Douala population (1.13% mortality without PBO and 25.81% after pre-exposure to PBO; *P* < 0.001), the Bertoua population (6.09% mortality without PBO and 18.15% after pre-exposure to PBO; *P* = 0.0174) and the Yaoundé population (57.88% mortality without PBO and 64.26% after pre-exposure to PBO; *P* = 0.5982). Similar results were also found with PBO + deltamethrin in the Douala population (9.44% mortality without PBO and 93.86% after pre-exposure to PBO; *P* < 0.001), the Bertoua population (22.07% mortality without PBO and 56.51% after pre-exposure to PBO; *P* = 0.001) and the Yaoundé population (63.57% mortality without PBO and 92.32% after pre-exposure to PBO; *P* = 0.001). Similar observations were made to alphacypermethrin in the three populations when pre-exposed to PBO. With pre-exposure to PBO, full recovery of susceptibility to bendiocarb was reported in all the populations tested: Bertoua population (65.36% mortality without PBO and 99.07% after pre-exposure to PBO; *P* < 0.001), Douala population (50.54% mortality without PBO and 100% after pre-exposure to PBO; *P* < 0.001) and Yaoundé population (51.68% mortality without PBO and 100% after pre-exposure to PBO; *P* < 0.001).

### WHO bottle test with clothianidin

**Fig. 3:**
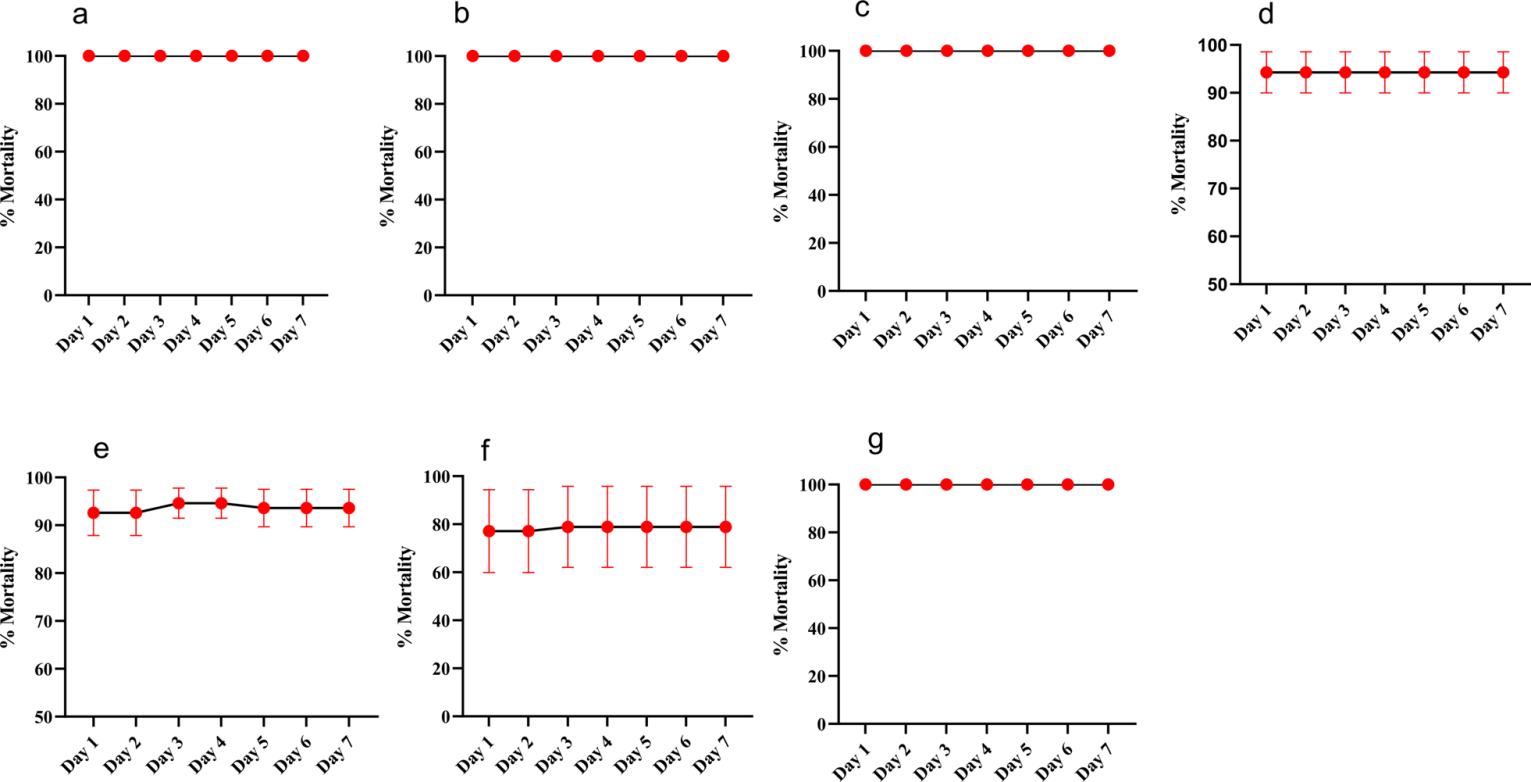
Mortality rate of adult *Aedes aegypti* 7 days after 1-h or 30-min exposure to clothianidin 20 µg/mL. **a**–**d**, mortality rates in Douala, Bertoua, Garoua and Yaoundé samples respectively; **e**–**g**, mortality rates 30 min after exposure in Douala, Bertoua and Garoua samples, respectively. Red dots, mortality rates; error bars represent standard deviation

Analyses were performed at two time points: 1-h or 30-min exposure using the discriminating dose of clothianidin (20 μg/mL). The results revealed that three of the four populations tested were full susceptible to clothianidin after 7 days observation (100% mortality in Bertoua, Douala and Garoua). However, the population from Yaoundé showed a probable resistance with a mortality rate of 94.26% (Fig. 3). To evaluate the efficacy of the insecticide as a function of exposure time, the exposure time was reduced to 30 min. The level of mortality varied according to localities. A total susceptibility was recorded in Garoua (100%); probable resistance was obtained in Douala with a mortality rate of 93.61%, while confirmed resistance was observed in Bertoua with a mortality rate of 78.88% (Fig. 3).

### Genotyping of three *kdr* mutations *F1534C*, *V410L* and *V1016I* in three towns in Cameroon

Mosquitoes resulting from exposure to permethrin and deltamethrin insecticides (live and dead) were used to assess the correlation between three *kdr* mutations, *F1534C*, *V1016I* and *V410L*, and resistance observed to the field samples in Yaoundé, Douala and Bertoua. The results are shown in Supplementary Material Tables S1, S2 and S3.

### Association between *F1534C kdr* mutation and pyrethroid resistance

The *F1534C* was found to be fixed in Douala in both groups of mosquitoes (dead and alive to deltamethrin and permethrin with allele frequency of 1.00) in Bertoua [(dead and alive to permethrin, alive to deltamethrin with allele frequency of 1.00) and almost-fixed in dead mosquitos exposed to deltamethrin with allelic frequency of 0.97]. The 1534C resistant allele was significantly associated with phenotypic resistance to deltamethrin and permethrin only in Yaoundé [odds ratio (OR) of 14.64; *P* < 0.001 and OR = ∞; *P* < 0.001, respectively, Fisher’s exact test] (Additional file 1 Fig. S1, Additional file 2 Table S1). No significant association was observed between *F1534C* genotypes and resistance to deltamethrin and permethrin in Bertoua.

### Association between *V410L kdr* mutation and pyrethroid resistance

Genotyping of mosquito for *V410L* mutation after insecticide testing of different populations revealed that susceptible homozygotes (VV) were abundant in both groups of mosquitoes (alive and dead) with allele frequencies ranging from 78.33% in alive deltamethrin in Yaoundé to 100% in dead deltamethrin from Douala and Bertoua (Additional file 3 Table S2). This mutation was found to be significantly associated with phenotypic resistance to deltamethrin in Yaoundé (OR = 2.538; *P* = 0.0327, Fisher’s exact test) and Douala (OR = ∞; *P* < 0.001 Fisher’s exact test). No significant association was observed between *V410L* genotypes and phenotype resistance to permethrin in Yaoundé and Douala (OR = 2.434; *P* = 0.3311 and OR = 1.511; *P* = 0.4986 respectively, Fisher’s exact test) as well as to neither permethrin nor deltamethrin in Bertoua (OR = 0.5745; *P* = 0.4353 and OR = 0; *P* = 0.2462 respectively, Fisher’s exact test) (Additional file 4 Fig. S2, Additional file 3 Table S2).

### Association between *V1016I kdr* mutation and pyrethroid resistance

Genotyping of mosquito for *V1016I* mutation after insecticide testing of different populations revealed that susceptible homozygotes (VV) were abundant in both groups of mosquitoes (alive and dead) with allele frequencies ranging from 63.33% in alive deltamethrin in Douala to 94,23% in dead deltamethrin in Douala (Additional file 5 Table S3). This mutation was significantly associated with phenotypic resistance to deltamethrin and permethrin in Yaoundé (OR = 2.842; *P* < 0.0136 and OR = 3.573; *P* = 0.0043 respectively, Fisher’s exact test) and Bertoua (OR = 3.295; *P* = 0.0017 and OR = 6.125; *P* < 0.001, respectively, Fisher’s exact test), to deltamethrin in Douala (OR = 3.201; *P* < 0.001, Fisher’s exact test). No significant association was observed between *V1016I* genotypes and phenotypic resistance to permethrin in Douala (OR = 2.071; *P* = 0.1400, Fisher’s exact test) (Additional file 6 Fig. S3, Additional file 5 Table S3).

### Combined effect of *F1534C*, *V410L* and *V1016I kdr* mutations in pyrethroid resistance in *Aedes aegypti* populations from Cameroon

Examining the three *kdr* mutations (1534 + 410 + 1016) in combination, 321 samples from the three localities were analysed, and we found six different genotypes: CC/LL/II, CC/VL/VI, FF/VV/VV, CC/VV/VV, FC/VV/VV and CC/VV/VI (Tables 1 and 2). The frequency of each genotype varied according to the location. The most common genotype was the single-mutant 1534 CC/VV/VV that was found in all groups of mosquitoes (alive and dead) with a frequency varying between 33% (dead permethrin Bertoua) and 100% (dead permethrin Douala). It was followed by the combination of CC/VV/VI genotype that was more present in the alive mosquitoes than the dead. This genotype was associated with resistance to deltamethrin in Douala (*P* = 0.0236) and Bertoua (*P* = 0.0307) and to permethrin in Yaoundé (*P* = 0.0011) and Bertoua (*P* = 0.0002) (Tables 2). The triple homozygous mutant CC/II/LL for the three mutations was present in all three localities tested but only in the live mosquitoes after exposure to deltamethrin or permethrin. It was associated with resistance to deltamethrin in Douala (*P* = 0.0064) (Tables 2). The triple homozygous wild-type FF/VV/VV was found only in dead mosquitoes from Yaoundé exposed to permethrin and deltamethrin.

**Table 1:** Different genotypes of *Aedes aegypti* populations in Yaoundé, Douala and Bertoua after exposure to deltamethrin and permethrin, considering *kdr F1534C*, *V410L* and *V1016I* mutations in the *VGSC*

Samples	Insecticide	Phenotypes	*n*	Frequency of the combined (*F1534C* + *V410L* + *V1016I*) genotypes					
				CC/LL/II	CC/VL/VI	FF/VV/VV	CC/VV/VV	FC/VV/VV	CC/VV/VI
Yaoundé	Deltamethrin	Alive	29	0	0.45	0	0.49	0.03	0.03
		Dead	30	0	0.2	0.17	0.53	0.1	0
	Permethrin	Alive	29	0.03	0	0	0.59	0	0.38
		Dead	30	0	0.13	0.03	0.64	0.2	0
Douala	Deltamethrin	Alive	30	0.2	0	0	0.47	0	0.33
		Dead	26	0	0	0	00.88	0	0.12
	Permethrin	Alive	30	0.13	0	0	0.8	0	0.07
		Dead	11	0	0	0	1	0	0
Bertoua	Deltamethrin	Alive	29	0.04	0	0	0.41	0	0.55
		Dead	30	0	0	0	0.77	0	0.23
	Permethrin	Alive	30	0.1	0	0	0.33	0	0.57
		Dead	17	0.06	0	0	0.94	0	0

*n* number of sample, *F* phenylalanine, *C* cysteine, *V* valine, *L* leucine, *I* isoleucine

**Table 2:** Association of combinations of *F1534C*, *V410L* and *V1016I kdr* mutations with pyrethroid resistance in *Aedes aegypti* populations from Cameroon

Samples	Insecticide	Deltamethrin			Permethrin		
	Genotypes	Phenotypes		Fisher exact *P*-value	Phenotypes		Fisher exact *P*-value
		Dead	Alive		Dead	Alive	
Yaoundé	CC/VV/VV	16	15	Reference	21	17	Reference
	CC/VL/VI	6	13	0.2420	4	0	0.1343
	CC/LL/II	0	0	NA	0	1	0.4615
	FF/VV/VV	6	0	0.0627	0	0	NA
	FC/VV/VV	2	0	0.4886	5	0	0.1390
	CC/VV/VI	0	1	> 0.9999	0	11	0.0011*
	*n*	30	29	NA	30	29	NA
Douala	CC/VV/VV	23	14	Reference	11	24	Reference
	CC/VL/VI	0	0	NA	0	0	NA
	CC/LL/II	0	6	0.0064*	0	4	0.3091
	FF/VV/VV	0	0	NA	0	0	NA
	FC/VV/VV	0	0	NA	0	0	NA
	CC/VV/VI	3	10	0.0236*	0	2	> 0.9999
	n	26	30	NA	11	30	NA
Bertoua	CC/VV/VV	22	13	Reference	16	12	Reference
	CC/VL/VI	0	0	NA	0	0	NA
	CC/LL/II	0	1	0.3889	1	3	0.3192
	FF/VV/VV	1	0	> 0.9999	0	0	NA
	FC/VV/VV	0	0	NA	0	0	AN
	CC/VV/VI	7	15	0.0307*	0	15	0.0002*
	*n*	30	29	NA	17	30	NA

*Significant difference. *NA* not applicable, *n* number of sample

### Combined effect of the *V410L* and *V1016I kdr* mutations in pyrethroid resistance in *Aedes aegypti* populations from Cameroon

The combination of *V410L* and *V1016I kdr* mutations were examined to assess their effect in *A. aegypti* samples from the three localities tested. A total of 321 samples were examined, and the results revealed the presence of 04 different genotypes: VV/VV, VL/VI, VV/VI and LL/II (Additional file 7 Fig. S4) (Table 3). The predominant genotype was the double homozygous wild type VV/VV found in all the groups of mosquitoes (live and dead) with a frequency between 40% (dead permethrin Bertoua) and 100% (dead permethrin Douala). The second most common genotype was VV/VI, also present in the three localities (more present in the live mosquitoes than the dead) with a frequency of between 3% (live deltamethrin Yaoundé) and 50% (live permethrin Bertoua). This genotype was found to be correlated with resistance observed to deltamethrin in Douala (*P* = 0.0236) and Bertoua (*P* = 0.0296) and to permethrin in Yaoundé (*P* = 0.0003) and Bertoua (*P* = 0.0012). The double mutant homozygous LL/II was present in all three localities tested and was found to be correlated with permethrin and deltamethrin resistance observed in Douala (*P* = 0.0064). The double heterozygous VL/VI genotype was found only in Yaoundé samples (dead permethrin and dead and live deltamethrin). This genotype also plays a role in deltamethrin resistance in this locality (*P* = 0.0497) (Table 3).

**Table 3:** Association between combination of *V410L* + *V1016I* mutations and phenotype resistance to deltamethrin and permethrin in *Aedes aegypti* populations from Cameroon

Samples	Insecticide	Deltamethrin			Permethrin		
	Genotypes	Phenotypes		Fisher exact *P*-value	Phenotypes		Fisher exact *P*-value
		Dead	Alive		Dead	Alive	
Yaoundé	VV/VV	24	15	Reference	26	17	Reference
	VL/VI	6	13	0.0497*	4	0	0.2814
	VV/VI	0	1	0.4000	0	11	0.0003*
	LL/II	0	0	NA	0	1	0.4091
	n	30	29	NA	30	29	NA
Douala	VV/VV	23	14	Reference	11	25	Reference
	VL/VI	0	0	NA	0	0	NA
	VV/VI	3	10	0.0236*	0	1	> 0.9999
	LL/II	0	6	0.0064*	0	4	0.5602
	n	26	30	NA	11	30	NA
Bertoua	VV/VV	23	13	Reference	15	12	Reference
	VL/VI	0	0	NA	0	0	NA
	VV/VI	7	15	0.0296*	1	15	0.0012*
	LL/II	0	1	0.3784	1	3	0.3326
	*n*	30	29	NA	17	30	NA

*Significant difference. *n* number of sample, *NA* not applicable

### Genetic diversity of *VGSC* in *Ae. aegypti*

The fragments of the *VGSC* gene covering the three *kdr* mutations 1534 (635 bp), 410 (500 bp) and 1016 (581 bp) were successfully sequenced in 107 *A. aegypti* (alive: 65 and dead: 42) from the 03 localities (Douala, Yaoundé and Bertoua) exposed to permethrin.

**Fig. 4:**
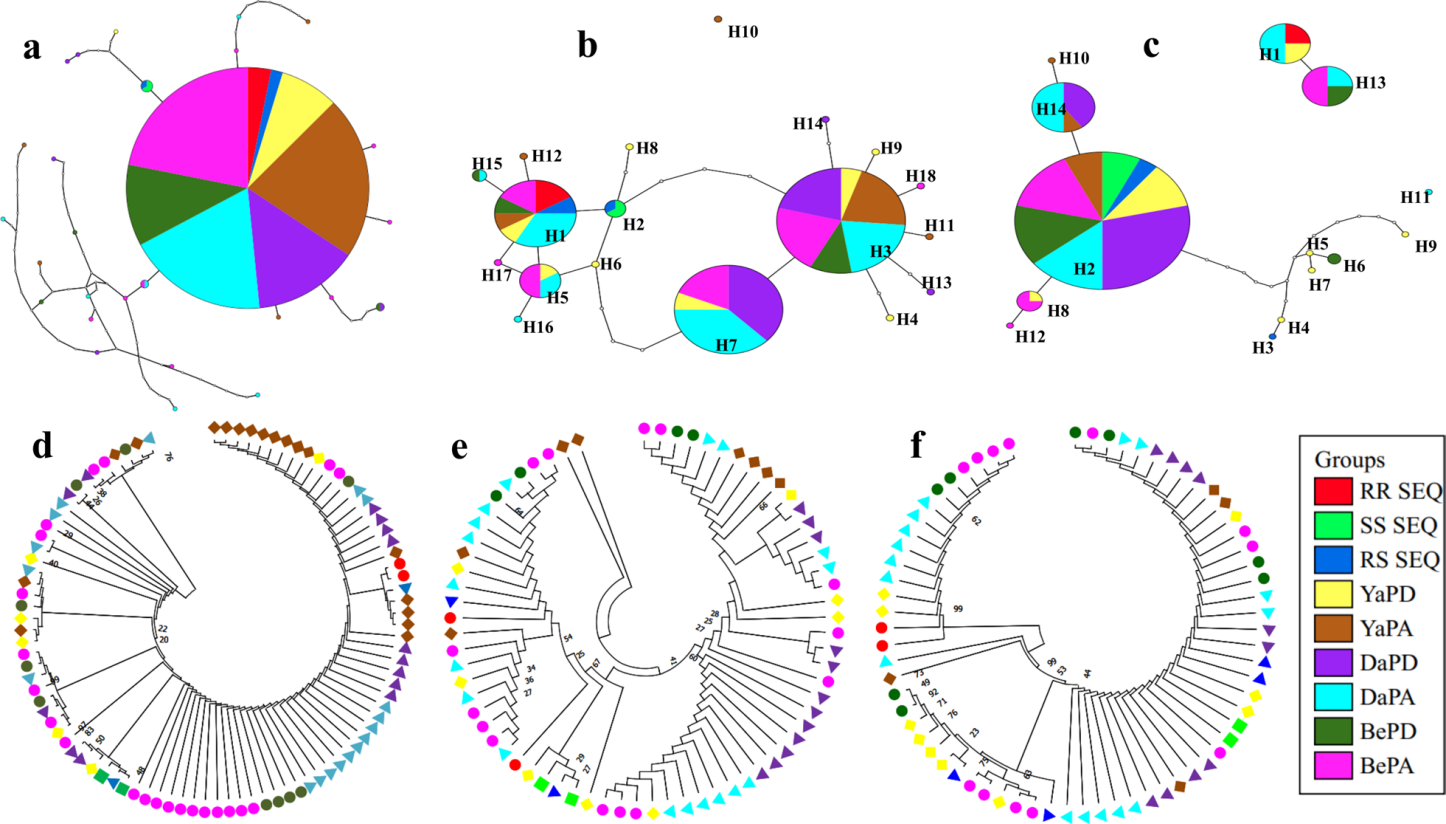
Pattern of genetic variability and polymorphism of the voltage-gated sodium channel in *Aedes aegypti*. Haplotype network for the *VGSC* sequence of the domains III (**a**), II (**b**) and I (**c**) taking into account different populations and resistance status. Phylogenetic tree of DNA sequences by maximum likelihood with the Jukes Cantor model of the domains III (**d**), II (**e**) and I (**f**); *RR SEQ* homozygote resistance sequence, *SS* homozygote susceptible sequence, *RS* heterozygote reference sequence, *YaDP* dead permethrin Yaoundé, *YaAP* alive permethrin Yaoundé, *DaDP* dead permethrin Douala, *DaAP* live permethrin Douala, *BeDP* dead permethrin Bertoua, *BeAP* alive permethrin Bertoua

For the domain covering the *F1534C* mutation, 44 sequences (live: 29 and dead: 15) were analysed. The analysis confirmed the presence of the 1534C mutation in all samples tested (Yaoundé, Douala and Bertoua). In total, 43 polymorphic sites, 21 haplotypes with low haplotype diversity (0.422) and low nucleotide diversity (0.00425) were found (Table 4). Among the haplotypes, one was the most represented (76.13%) (Fig. 4a). A maximum likelihood (ML) tree of the sequences analysed confirms a high diversity, with six probable clusters (Fig. 4b). Overall, all the estimated statistics were negative (*D* = −1.90437, Fs Fu = −15.737) with Fs Fu being statistically significant (Table 4).

**Table 4:** Genetic diversity parameters of *F1534C*, *V410L* and *V1016I kdr* mutation of *Aedes aegypti* populations in Yaoundé, Douala and Bertoua

*F1534C kdr* mutation										
Samples	2N	*S*	Syn	Nsyn	**π**	*H*	Hd	*D*	Fu Fs	*P*
YaPD	6	6	2	3	0.0067	3	0.6	0.37522	1.672	> 0.10
YaPA	18	27	13	14	0.00882	5	0.405	−2.11617	2.386	< 0.05
DaPD	14	27	10	17	0.01357	6	0.604	−1.41668	1.919	> 0.10
DaPA	18	22	6	12	0.00902	7	0.569	−1.62506	0.423	> 0.05
BePD	10	7	1	4	0.00547	4	0.533	−0.77402	0.617	> 0.10
BePA	22	15	9	7	0.00533	9	0.606	−1.78737	−2.569	> 0.05
Total	88	43	6	11	0.00425	21	0.422	−1.90437	−15.737	< 0.05
*V410L kdr* mutation										
YaPD	8	11	2	8	0.01197	8	1	0.30321	−4.234	*P* > 0.10
YaPA	8	16	3	14	0.01425	5	0.786	−0.94356	0.941	*P* > 0.10
DaPD	12	5	0	4	0.00322	4	0.682	−0.98759	−0.207	*P* > 0.10
DaPA	18	8	0	7	0.00895	6	0.817	1.55423	1.018	*P* > 0.10
BePA	4	6	0	6	0.0102	3	0.833	1.66214	1.099	*P* > 0.10
BePA	14	7	0	6	0.009	6	0.857	1.30262	0.417	*P* > 0.10
Total	70	25	0	0	0.00958	18	0.844	−1.19891	−4.125	*P* > 0.10
*V1016I kdr* mutation										
YaPD	10	59	9	42	0.05725	7	0.911	0.12648	2.826	*P* > 0.10
YaPA	4	1	0	1	0.00132	2	0.5	−0.61237	0.172	*P* > 0.10
DaPD	12	0	0	0	0	0	0	0	0	NA
DaPA	16	55	16	36	0.06662	3	0.575	1.78916	20.093	*P* > 0.05
BePD	8	54	11	38	0.06219	3	0.714	0.71032	10.545	*P* > 0.10
BePA	12	50	12	35	0.05709	4	0.782	1.26302	10.404	*P* > 0.10
Total	68	68	0	0	0.05137	12	0.636	0.93917	16.025	*P* > 0.10

*2N* number of sequences, *S* number of polymorphic sites, *h* number of haplotypes, *Hd* haplotype diversity, *π* nucleotide diversity, *Syn and Nsyn* synonymous and non-synonymous mutation, *D and Fs* Tajima’s D and Fu Fs statistics, *YaDP* dead permethrin Yaoundé, *YaAP* live permethrin Yaoundé, *DaDP* dead permethrin Douala, *DaAP* live permethrin Douala, *BeDP* dead permethrin Bertoua, *BeAP* live permethrin Bertoua, *NA* not applicable

Analysis of 32 sequences of the part of the gene covering codon I revealed a high degree of polymorphism with 18 haplotypes, a haplotype diversity of 0.844 and a nucleotide diversity of 0.00958 (Table 4). Haplotypes H1 (12.85%), H3 (27.14%), H5 (8.57%) and H7 (22.28%) were the most prevalent and mainly found in live mosquitoes (Fig. 4c). Overall, all the estimated statistics were negative (Tajima *D* = −1.19891 and Fu Fs = −4.125) (Table 4). The maximum likelihood (ML) tree of the sequences analysed confirms a high diversity with four probable clusters (Fig. 4d).

Similarly, the analysis of 31 sequences of the portion of the gene covering codon II showed a high polymorphism with 14 haplotypes, a haplotypic diversity of 0.636 and a nucleotide diversity of 0.05137 (see Table 4). The most widespread haplotypes, H1 (25.80%) and H2 (61.29%), were mainly found in live mosquitoes (Fig. 4e). Overall, all of the estimated statistics were positive (Tajima *D* = 0.93917 and Fu Fs = 16.025) (Table 4). The maximum likelihood (ML) tree of the sequences analysed confirms a high diversity with four probable clusters (Fig. 4f).

## Discussion

### Insecticide resistance profile of *Aedes aegypti*

This study investigated the distribution and role of three *kdr* mutations in conferring pyrethroid resistance in *A. aegypti*, in Cameroon. The results show that *A. aegypti* is resistant to pyrethroids and carbamate insecticides but remains susceptible to organophosphates. These observations are consistent with the previous studies carried out in Cameroon [29–33, 51], elsewhere in Africa [51–56] and outside Africa [39]. The resistance observed in *A. aegypti* to these insecticides (deltamethrin, permethrin, alphacypermethrin and bendiocarb) is difficult to explain because regular insecticidal control for *Aedes* mosquitoes is very limited in Central Africa. However, the use of organophosphate and bendiocarb to control *A. aegypti* was reported in Sao Tome and Principe Island, located in Central Africa [57]. Nevertheless, the question of the origin of the selective pressure in this mosquito species in certain African countries remains. Some authors hypothesise that the resistance observed in *A. aegypt*i could be due to the fact that this species thriving to urban environments, which are more exposed to pollution and domestic exposure to insecticides through indoor spraying and impregnated bed nets used for malaria control [28]. In addition, the use of pesticides and fertilisers in agriculture to protect and grow vegetable crops could also promote the emergence of resistance in mosquitoes by contaminating their breeding and resting sites as has been suggested [51].

A partial or full recovery of susceptibility observed in the different populations of *A. aegypti* to permethrin, deltamethrin, alphacypermethrin and bendiocarb after pre-exposure to the PBO synergist suggests that cytochrome P450 monooxygenases play an important role in the resistance observed which is consistent with previous data from Central Africa [29, 30, 32, 52, 58] and elsewhere [39]. This observation shows that adding PBO to pyrethroids or carbamate for ultra-low volume (ULV) application will be more effective for *Aedes* control.

The bioassays carried out with the new class of insecticide being tested, introduced more recently (early 2020) for malaria control in Africa [59] revealed that all populations tested were susceptible to clothianidin. These results corroborate previous observations in Mexico [60] and suggest that this insecticide can be used as one alternative for chemical control of *Aedes* mosquitoes in Cameroon.

### Association between pyrethroid resistance and *kdr* mutations

This study showed that the *F1534C*, *V410L* and *V1016I kdr* mutations are present in *A. aegypti* populations in Cameroon and coexist in some locations. The presence of these mutations has been previously reported in Cameroon [29, 32] and other countries in Africa [28, 61]. The *F1534C* that is the most widespread mutation in *A. aegypti* was found to be associated to deltamethrin and permethrin resistance in Yaoundé. However, the fact that this mutation tends to be fixed in other localities (Douala and Bertoua) did not allow for the investigation of its implication in the observed resistance. This fixation of *F1534C* mutation observed aligns with previous research conducted in the same locations in Cameroon, indicating that this mutation has been present for some time and is gradually spreading [29, 32]. In the space of 5 years, the frequency of this mutation has risen from 33.33% in Douala and 0% in Yaoundé in 2017 [31] to 88% in Douala and 7% in Yaoundé in 2021 [32] respectively. The *V410L* mutation was found to be associated to deltamethrin resistance in Douala and Yaoundé, while *V1016I* mutation was correlated to deltamethrin and permethrin resistance in Yaoundé and Bertoua. In comparison with the findings of previous studies conducted in Cameroon [32], an increase in the frequency of both mutations was observed in this study. For example, the 1016I allele increased from 0.1 to 0.36 in Douala and from 0.07 to 0.24 in Yaoundé. This finding indicates the subsequent dissemination of these mutations following their introduction into Cameroon.

When examining combinations of the three *kdr* mutations (*F1534C*, *V1016I* and *V410L*), six genotypes were found. The low number of genotypes observed could be due to the fixation of the *F1534C*. This observation is consistent with the results obtained in Burkina Faso [62] but differs from those previously observed in Cameroon when 14 different genotypes were identified [29] and in other parts of the world where a high diversity of genotypes has been recorded, particularly in Harris County, USA [63], in Mexico [42], in Colombia [64] and Luanda, Zambia [53], with 23, 20, 14 and 12 genotypes respectively. The triple homozygous mutant CC/II/LL was present in live mosquitoes after exposure to deltamethrin or permethrin in all the three localities tested in Cameroon, showing the implication of the triple-homozygote-resistant genotype in the pyrethroid resistance observed in these populations. Similar findings have been reported in Niger [28] and in Mexico [42], whereas in Ghana, this tri-locus genotype has been only associated with permethrin resistance [65].

Considering *V410L* and *V1016I*, a dual effect of these mutations was observed in the resistance to pyrethroids in the populations tested. The double homozygote LL/II was found to be associated with deltamethrin and permethrin resistance in Douala, while the double heterozygote VL/VI was associated with deltamethrin resistance only in Yaoundé. A similar observation was made in other African countries, particularly in Niger [28] and Burkina Faso [62], as well as in South America [42].

The observed negative values of the Tajima’s D and Fu Fs indexes, coupled with high haplotype diversity and low nucleotide diversity, confirm the recent spread of these mutations across *A. aegypti* populations in Cameroon. This suggests a rapid increase in the frequency of resistance alleles, possibly due to strong selective pressure from the intensive use of pyrethroid- and carbamate-based insecticides, an unintended consequence of the widespread use of insecticides in agriculture and malaria control.

Individually or in combination, these three *kdr* mutations have been shown to be associated with the pyrethroid resistance observed in Cameroon. This situation could have implications for vector control.

This study has the limitation that the association between the three *kdr* mutations and pyrethroid resistance was not assessed in the Garoua samples. However, a previous study in Cameroon revealed that the F1534C mutation was absent in Garoua samples [32]. Taking into account the dynamics of resistance, further studies investigating the presence and association of these mutations in different ecological zones in Cameroon, including Garoua, are needed.

## Conclusions

The study result revealed that *A. aegypti* populations from some locations in Cameroon are resistant to pyrethroids and carbamates but susceptible to organophosphates and neonicotinoids. The two latter insecticide classes are suitable for the control of *A. aegypti* in these localities. A full or partial recovery of susceptibility observed after pre-exposure of mosquitoes to PBO suggests a role of P450 genes in the resistance observed, particularly to bendiocarb. *F1534C*, *V410L* and *V1016I* were found to be associated with pyrethroid resistance observed with allelic frequencies increasing over the time. This study provides important data that could help to develop effective strategies to control *A. aegypti* arbovirus vectors in Cameroon. Indeed, the resistance pattern to insecticides found combined with the resistance mechanism involved enables the country to select the most effective molecule to implement insecticide-based control interventions in case of outbreak.

## Supplementary Information

Additional file 1.

Additional file 2.

Additional file 3.

Additional file 4.

Additional file 5.

## Data Availability

Data are provided within the manuscript or supplementary information files.

## Abbreviations

*A*: *Aedes*
Ace: Acetylcholinesterase the *GABA*
DNTp: Deoxynucleoside triphosphates
*kdr*: Knock down resistance gene
MgCl: Magnesium chloride
OR: Odds ratio
PCR: Polymerase chain reaction
UK: United Kingdom
ULV: Ultra low volume
UV light: Ultraviolet

## References

[1] Bhatt S, Gething PW, Brady OJ, Messina JP, Farlow AW, Moyes CL, et al. The global distribution and burden of dengue. Nature. 2013;496:504–7. 23563266 10.1038/nature12060 PMC3651993

[2] Paz-Bailey G, Adams LE, Deen J, Anderson KB, Katzelnick LC. Dengue. Lancet. 2024;403:667–82. 38280388 10.1016/S0140-6736(23)02576-X PMC12372472

[3] Dalpadado R, Amarasinghe D, Gunathilaka N, Ariyarathna N. Bionomic aspects of dengue vectors *Aedes aegypti* and *Aedes albopictus* at domestic settings in urban, suburban and rural areas in Gampaha District, Western Province of Sri Lanka. Parasit Vectors. 2022;15:148. 35477476 10.1186/s13071-022-05261-3 PMC9044863

[4] Dzul-Manzanilla F, Ibarra-López J, Bibiano Marín W, Martini-Jaimes A, Leyva JT, Correa-Morales F, et al. Indoor resting behavior of *Aedes aegypti* (diptera: Culicidae) in Acapulco, Mexico. J Med Entomol. 2017;54:501–4. 28011725 10.1093/jme/tjw203

[5] Bitsindou P, Bantsimba-Ndziona M, Lenga A. Distribution actuelle et caractérisations bioécologiques d’*Aedes aegypti* et d’*Aedes albopictus* dans deux arrondissements de Brazzaville. Bulletin de la Société de Pathologie Exoique. 2018;111:301–8. 10.3166/bspe-2019-0056 30950593

[6] Kamgang B, Vazeille M, Tedjou AN, Wilson-Bahun TA, Yougang AP, Mousson L, et al. Risk of dengue in Central Africa: vector competence studies with *Aedes aegypti* and *Aedes albopictus* (Diptera: Culicidae) populations and dengue 2 virus. PLoS Negl Trop Dis. 2019;13:e0007985. 31887138 10.1371/journal.pntd.0007985 PMC6953884

[7] Kamgang B, Vazeille M, Tedjou A, Yougang AP, Wilson-Bahun TA, Mousson L, et al. Different populations of *Aedes aegypti* and *Aedes albopictus* (Diptera: Culicidae) from Central Africa are susceptible to Zika virus infection. PLOS Negl Trop Dis. 2020;14:e0008163. 10.1371/journal.pntd.0008163. 32203510 10.1371/journal.pntd.0008163 PMC7117767

[8] Kamgang B, Vazeille M, Yougang AP, Tedjou AN, Wilson-Bahun TA, Mousson L, et al. Potential of *Aedes albopictus* and *Aedes aegypti* (Diptera: Culicidae) to transmit yellow fever virus in urban areas in Central Africa. Emerg Microbes Infect. 2019;8:1636–41. 31711378 10.1080/22221751.2019.1688097 PMC6853216

[9] WHO. Pesticides and their application: for the control of vectors and pests of public health importance. Geneva: World Health Organization; 2006.

[10] Kroeger A, Lenhart A, Ochoa M, Villegas E, Levy M, Alexander N, et al. Effective control of dengue vectors with curtains and water container covers treated with insecticide in Mexico and Venezuela: cluster randomised trials. BMJ. 2006;332:1247–52. 16735334 10.1136/bmj.332.7552.1247 PMC1471956

[11] Becker N, Ludwig M, Su T. Lack of resistance in *Aedes vexans* field populations after 36 years of *Bacillus thuringiensis* subsp. israelensis applications in the Upper Rhine Valley, Germany. J Am Mosquito Control Assoc. 2018;34:154–7. 10.2987/17-6694.1 31442151

[12] Marcombe S, Darriet F, Tolosa M, Agnew P, Duchon S, Etienne M, et al. Pyrethroid resistance reduces the efficacy of space sprays for dengue control on the island of Martinique (Caribbean). PLoS Negl Trop Dis. 2011;5:e1202. 21713017 10.1371/journal.pntd.0001202 PMC3119637

[13] Al-Amin HM, Gyawali N, Graham M, Alam MS, Lenhart A, Hugo LE, et al. Insecticide resistance compromises the control of *Aedes aegypti* in Bangladesh. Pest Manag Sci. 2023;79:2846–61. 36942761 10.1002/ps.7462 PMC11694321

[14] Ranson H, Burhani J, Lumjuan N, Black IV WC. Insecticide resistance in dengue vectors. TropIKA net [online]. 2009;1:1.

[15] Moyes CL, Vontas J, Martins AJ, Ng LC, Koou SY, Dusfour I, et al. Contemporary status of insecticide resistance in the major *Aedes* vectors of arboviruses infecting humans. PLoS Negl Trop Dis. 2017;11:e0005625. 28727779 10.1371/journal.pntd.0005625 PMC5518996

[16] Corbel V, Achee NL, Chandre F, Coulibaly MB, Dusfour I, Fonseca DM, et al. Tracking insecticide resistance in mosquito vectors of arboviruses: the worldwide insecticide resistance network (WIN). PLoS Negl Trop Dis. 2016;10:e0005054. 27906961 10.1371/journal.pntd.0005054 PMC5131894

[17] Vontas J, Kioulos E, Pavlidi N, Morou E, Della Torre A, Ranson H. Insecticide resistance in the major dengue vectors *Aedes albopictus* and *Aedes aegypti*. Pestic Biochem Physiol. 2012;104:126–31.

[18] Corbel V, Kont MD, Ahumada ML, Andréo L, Bayili B, Bayili K, et al. A new WHO bottle bioassay method to assess the susceptibility of mosquito vectors to public health insecticides: results from a WHO-coordinated multi-centre study. Parasit Vectors. 2023;16:21. 36670470 10.1186/s13071-022-05554-7 PMC9863080

[19] Samal RR, Kumar S. Cuticular thickening associated with insecticide resistance in dengue vector, *Aedes aegypti* L. Int J Trop Insect Sci. 2021;41:809–20.

[20] Jacobs E, Chrissian C, Rankin-Turner S, Wear M, Camacho E, Broderick NA, et al. Cuticular profiling of insecticide resistant *Aedes aegypti*. Sci Rep. 2023;13:10154. 37349387 10.1038/s41598-023-36926-3 PMC10287657

[21] Paeporn P, Supaphathom K, Sathantriphop S, Chareonviritaphap T, Yaicharoen R. Behavioural responses of deltamethrin-and permethrin-resistant strains of *aedes aegypti* when exposed to permethrin in an excito-repellency test system. 2007.

[22] Ishak IH, Riveron JM, Ibrahim SS, Stott R, Longbottom J, Irving H, et al. The Cytochrome P450 gene CYP6P12 confers pyrethroid resistance in kdr-free Malaysian populations of the dengue vector *Aedes albopictus*. Sci Rep. 2016;6:24707. 27094778 10.1038/srep24707 PMC4837359

[23] Daborn PJ, Le Goff G. The genetics and genomics of insecticide resistance. Trends Genet. 2004;20:163–70. 15036810 10.1016/j.tig.2004.01.003

[24] Sombie A, Saiki E, Yameogo F, Sakurai T, Shirozu T, Fukumoto S, et al. High frequencies of F1534C and V1016I kdr mutations and association with pyrethroid resistance in *Aedes aegypti* from Somgande (Ouagadougou), Burkina Faso. Trop Med Health. 2019;47:2. 10.1186/s41182-018-0134-5. 30787670 10.1186/s41182-018-0134-5 PMC6318976

[25] Kushwah RBS, Dykes CL, Kapoor N, Adak T, Singh OP. Pyrethroid-resistance and presence of two knockdown resistance (kdr) mutations, F1534C and a novel mutation T1520I, in Indian *Aedes aegypti*. PLoS Negl Trop Dis. 2015;9:e3332. 25569164 10.1371/journal.pntd.0003332 PMC4287524

[26] Haddi K, Tomé HV, Du Y, Valbon WR, Nomura Y, Martins GF, et al. Detection of a new pyrethroid resistance mutation (V410L) in the sodium channel of *Aedes aegypti*: a potential challenge for mosquito control. Sci Rep. 2017;7:46549. 28422157 10.1038/srep46549 PMC5396194

[27] Akhir MAM, Wajidi MFF, Lavoué S, Azzam G, Jaafar IS, Awang Besar NAU, et al. Knockdown resistance (kdr) gene of Aedes aegypti in Malaysia with the discovery of a novel regional specific point mutation A1007G. Parasit Vectors. 2022;15:122. 10.1186/s13071-022-05192-z. 35387654 10.1186/s13071-022-05192-z PMC8988349

[28] Maiga A-A, Sombié A, Zanré N, Yaméogo F, Iro S, Testa J, et al. First report of V1016I, F1534C and V410L kdr mutations associated with pyrethroid resistance in *Aedes aegypti* populations from Niamey, Niger. PLoS ONE. 2024;19:e0304550. 38809933 10.1371/journal.pone.0304550 PMC11135682

[29] Yougang AP, Keumeni CR, Wilson-Bahun TA, Tedjou AN, Njiokou F, Wondji C, et al. Spatial distribution and insecticide resistance profile of *Aedes aegypti* and *Aedes albopictus* in Douala, the most important city of Cameroon. PLoS ONE. 2022;17:e0278779. 36512581 10.1371/journal.pone.0278779 PMC9746985

[30] Yougang AP, Kamgang B, Tedjou AN, Wilson-Bahun TA, Njiokou F, Wondji CS. Nationwide profiling of insecticide resistance in *Aedes albopictus* (Diptera: Culicidae) in Cameroon. PLoS ONE. 2020;15:e0234572. 32555588 10.1371/journal.pone.0234572 PMC7302487

[31] Yougang AP, Kamgang B, Bahun TAW, Tedjou AN, Nguiffo-Nguete D, Njiokou F, et al. First detection of F1534C knockdown resistance mutation in *Aedes* aegypti (Diptera: Culicidae) from Cameroon. Infect Dis Poverty. 2020;9:1–12. 33138860 10.1186/s40249-020-00769-1 PMC7607635

[32] Montgomery M, Harwood JF, Yougang AP, Wilson-Bahun TA, Tedjou AN, Keumeni CR, et al. Spatial distribution of insecticide resistant populations of *Aedes aegypti* and *Ae. albopictus* and first detection of V410L mutation in *Ae. aegypti* from Cameroon. Infect Dis Poverty. 2022;11:1–13. 35974351 10.1186/s40249-022-01013-8 PMC9382841

[33] Kamgang B, Yougang AP, Tchoupo M, Riveron JM, Wondji C. Temporal distribution and insecticide resistance profile of two major arbovirus vectors *Aedes aegypti* and *Aedes albopictus* in Yaounde, the capital city of Cameroon. Parasit Vectors. 2017;10:469. 10.1186/s13071-017-2408-x. 29017606 10.1186/s13071-017-2408-x PMC5635539

[34] Djiappi-Tchamen B, Nana-Ndjangwo MS, Mavridis K, Talipouo A, Nchoutpouen E, Makoudjou I, et al. Analyses of insecticide resistance genes in *Aedes aegypti* and *Aedes albopictus* mosquito populations from Cameroon. Genes. 2021;12:828. 34071214 10.3390/genes12060828 PMC8229692

[35] Jupp PG. Mosquitoes of Southern Africa: culicinae and toxorhynchitinae. Hartbeespoort: Ekogilde Publishers; 1996.

[36] Edwards F. Mosquitoes of the Ethiopian Region: Culicine Adults and Pupae. Mosquitoes of the Ethiopian Region: Culicine Adults and Pupae. 1941.

[37] Marcombe S, Mathieu RB, Pocquet N, Riaz M-A, Poupardin R, Sélior S, et al. Insecticide resistance in the dengue vector *Aedes aegypti* from Martinique: distribution, mechanisms and relations with environmental factors. PLoS ONE. 2012;7:e30989. 22363529 10.1371/journal.pone.0030989 PMC3283601

[38] WHO. Manual for monitoring insecticide resistance in mosquito vectors and selecting appropriate interventions. 2022.

[39] Ishak IH, Jaal Z, Ranson H, Wondji CS. Contrasting patterns of insecticide resistance and knockdown resistance (kdr) in the dengue vectors *Aedes aegypti* and *Aedes albopictus* from Malaysia. Parasit Vectors. 2015;8:1–13. 25888775 10.1186/s13071-015-0797-2 PMC4377062

[40] Livak KJ. Organization and mapping of a sequence on the Drosophila melanogaster X and Y chromosomes that is transcribed during spermatogenesis. Genetics. 1984;107:611–34. 6430749 10.1093/genetics/107.4.611 PMC1202380

[41] Saavedra-Rodriguez K, Urdaneta-Marquez L, Rajatileka S, Moulton M, Flores A, Fernandez-Salas I, et al. A mutation in the voltage-gated sodium channel gene associated with pyrethroid resistance in Latin American *Aedes aegypti*. Insect Mol Biol. 2007;16:785–98. 18093007 10.1111/j.1365-2583.2007.00774.x

[42] Saavedra-Rodriguez K, Maloof FV, Campbell CL, Garcia-Rejon J, Lenhart A, Penilla P, et al. Parallel evolution of vgsc mutations at domains IS6, IIS6 and IIIS6 in pyrethroid resistant *Aedes aegypti* from Mexico. Sci Rep. 2018;8:6747. 29712956 10.1038/s41598-018-25222-0 PMC5928250

[43] Leong C-S, Vythilingam I, Liew JW-K, Wong M-L, Wan-Yusoff WS, Lau Y-L. Enzymatic and molecular characterization of insecticide resistance mechanisms in field populations of *Aedes aegypti* from Selangor, Malaysia. Parasit Vectors. 2019;12:1–17. 31097010 10.1186/s13071-019-3472-1 PMC6521414

[44] Hall T. BioEdit: a user-friendly biological sequence alignment editor and analysis program for Windows 95/98/NT. In: Nucleic acids symposium series: 1999. [London]: Information Retrieval Ltd. 1999; 1c1979-c2000:e0007615.

[45] Rozas J, Ferrer-Mata A, Sánchez-DelBarrio JC, Guirao-Rico S, Librado P, Ramos-Onsins SE, et al. DnaSP 6: DNA sequence polymorphism analysis of large data sets. Mol Biol Evol. 2017;34:3299–302. 29029172 10.1093/molbev/msx248

[46] Tajima F. Statistical method for testing the neutral mutation hypothesis by DNA polymorphism. Genetics. 1989;123:585–95. 2513255 10.1093/genetics/123.3.585 PMC1203831

[47] Fu Y-X. Statistical tests of neutrality of mutations against population growth, hitchhiking and background selection. Genetics. 1997;147:915–25. 9335623 10.1093/genetics/147.2.915 PMC1208208

[48] Tamura K, Stecher G, Peterson D, Filipski A, Kumar S. MEGA6: molecular evolutionary genetics analysis version 6.0. Mol Biol Evol. 2013;30:2725–9. 24132122 10.1093/molbev/mst197 PMC3840312

[49] Clement M, Posada D, Crandall KA. TCS: a computer program to estimate gene genealogies. Mol Ecol. 2000;9:1657–9. 11050560 10.1046/j.1365-294x.2000.01020.x

[50] dos Santos A, Cabezas M, Tavares A, Xavier R, Mii B. tcsBU: a tool to extend TCS network layout and visualization. Bioinformatics. 2016;32:627–8. 26515821 10.1093/bioinformatics/btv636

[51] Kamgang B, Marcombe S, Chandre F, Nchoutpouen E, Nwane P, Etang J, et al. Insecticide susceptibility of Aedes aegypti and *Aedes albopictus* in Central Africa. Parasit Vectors. 2011;4:1–8. 21575154 10.1186/1756-3305-4-79 PMC3121691

[52] Kamgang B, Wilson-Bahun TA, Yougang AP, Lenga A, Wondji CS. Contrasting resistance patterns to type I and II pyrethroids in two major arbovirus vectors *Aedes aegypti* and *Aedes albopictus* in the Republic of the Congo, Central Africa. Infect Dis Poverty. 2020;9:23. 10.1186/s40249-020-0637-2. 32114983 10.1186/s40249-020-0637-2 PMC7050138

[53] Ayres CF, Seixas G, Borrego S, Marques C, Monteiro I, Marques CS, et al. The V410L knockdown resistance mutation occurs in island and continental populations of *Aedes aegypti* in West and Central Africa. PLoS Negl Trop Dis. 2020;14:e0008216. 32384079 10.1371/journal.pntd.0008216 PMC7304628

[54] Badolo A, Sombié A, Pignatelli PM, Sanon A, Yaméogo F, Wangrawa DW, et al. Insecticide resistance levels and mechanisms in *Aedes aegypti* populations in and around Ouagadougou, Burkina Faso. PLoS Negl Trop Dis. 2019;13:e0007439. 31120874 10.1371/journal.pntd.0007439 PMC6550433

[55] Sene NM, Mavridis K, Ndiaye EH, Diagne CT, Gaye A, Ngom EHM, et al. Insecticide resistance status and mechanisms in *Aedes aegypti* populations from Senegal. PLoS Negl Trop Dis. 2021;15:e0009393. 33970904 10.1371/journal.pntd.0009393 PMC8136859

[56] Kwame Amlalo G, Akorli J, Etornam Akyea-Bobi N, Sowa Akporh S, Aqua-Baidoo D, Opoku M, et al. Evidence of high frequencies of insecticide resistance mutations in *Aedes aegypti* (Culicidae) mosquitoes in urban Accra, Ghana: implications for insecticide-based vector control of *Aedes*-borne Arboviral diseases. J Med Entomol. 2022;59:2090–101. 36066455 10.1093/jme/tjac120

[57] Kamgang B, Acântara J, Tedjou A, Keumeni C, Yougang A, Ancia A, et al. Entomological surveys and insecticide susceptibility profile of *Aedes aegypti* during the dengue outbreak in Sao Tome and Principe in 2022. PLoS Negl Trop Dis. 2024;18:e0011903. 38829904 10.1371/journal.pntd.0011903 PMC11175431

[58] Ngoagouni C, Kamgang B, Brengues C, Yahouedo G, Paupy C, Nakouné E, et al. Susceptibility profile and metabolic mechanisms involved in *Aedes aegypti* and *Aedes albopictus* resistant to DDT and deltamethrin in the Central African Republic. Parasit Vectors. 2016;9:1–13. 27881148 10.1186/s13071-016-1887-5 PMC5121976

[59] Odjo EM, Akpodji CS, Djènontin A, Salako AS, Padonou GG, Adoha CJ, et al. Did the prolonged residual efficacy of clothianidin products lead to a greater reduction in vector populations and subsequent malaria transmission compared to the shorter residual efficacy of pirimiphos-methyl? Malar J. 2024;23:119. 38664703 10.1186/s12936-024-04949-4 PMC11047034

[60] Che-Mendoza A, González-Olvera G, Medina-Barreiro A, Arisqueta-Chablé C, Herrera-Bojórquez J, Bibiano-Marín W, et al. Residual efficacy of the neonicotinoid insecticide clothianidin against pyrethroid-resistant *Aedes aegypti*. Pest Manag Sci. 2023;79:638–44. 10.1002/ps.7231. 36223080 10.1002/ps.7231 PMC9845138

[61] Toé HK, Zongo S, Guelbeogo MW, Kamgang B, Viana M, Tapsoba M, et al. Multiple insecticide resistance and first evidence of V410L kdr mutation in *Aedes* (Stegomyia) *aegypti* (Linnaeus) from Burkina Faso. Med Vet Entomol. 2022;36:309–19. 35869781 10.1111/mve.12602

[62] Sombié A, Ouédraogo WM, Oté M, Saiki E, Sakurai T, Yaméogo F, et al. Association of 410L, 1016I and 1534C kdr mutations with pyrethroid resistance in *Aedes aegypti* from Ouagadougou, Burkina Faso, and development of a one-step multiplex PCR method for the simultaneous detection of 1534C and 1016I kdr mutations. Parasit Vectors. 2023;16:137. 37076920 10.1186/s13071-023-05743-y PMC10116651

[63] Hernandez JR, Liu S, Fredregill CL, Pietrantonio PV. Impact of the V410L kdr mutation and co-occurring genotypes at kdr sites 1016 and 1534 in the VGSC on the probability of survival of the mosquito *Aedes aegypti* (L.) to Permanone in Harris County, TX, USA. PLoS Negl Trop Dis. 2023;17:e0011033. 36689414 10.1371/journal.pntd.0011033 PMC9870149

[64] Granada Y, Mejía-Jaramillo AM, Strode C, Triana-Chavez O. A point mutation V419L in the sodium channel gene from natural populations of *Aedes aegypti* is involved in resistance to λ-cyhalothrin in Colombia. Insects. 2018;9:23. 29443870 10.3390/insects9010023 PMC5872288

[65] Abdulai A, Owusu-Asenso CM, Akosah-Brempong G, Mohammed AR, Sraku IK, Attah SK, et al. Insecticide resistance status of *Aedes aegypti* in southern and northern Ghana. Parasit Vectors. 2023;16:135. 37072865 10.1186/s13071-023-05752-x PMC10111668

