# Supplementary figures and images for: Association of knockdown resistance mutations with pyrethroid resistance in Aedes aegypti, a major arbovirus vector in Cameroon
Source: Parasit Vectors. 2025 Jul 24;18:296. doi: 10.1186/s13071-025-06943-4 (PMC12291522; doi:10.1186/s13071-025-06943-4)

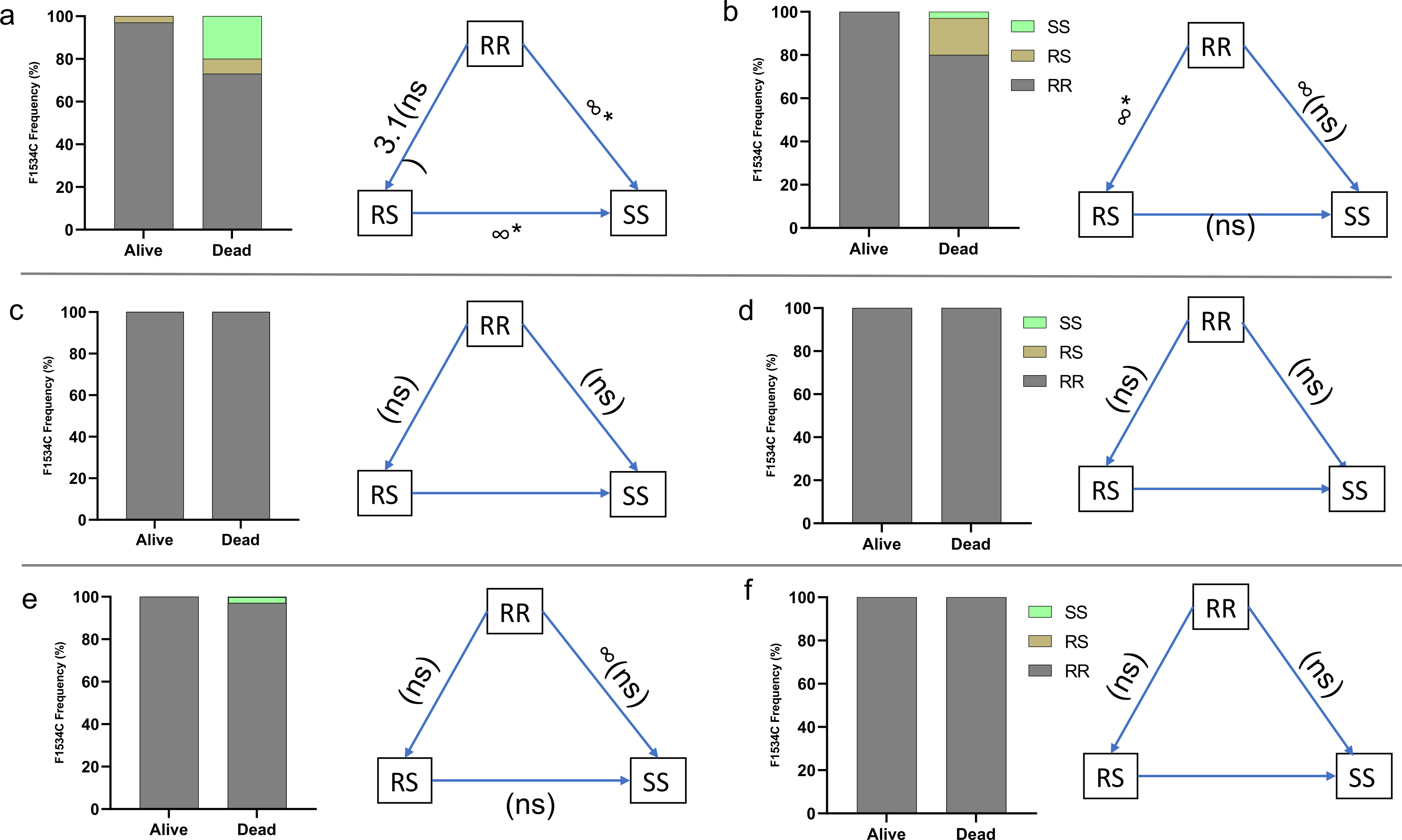

Supplement: Supplementary file 1 — Additional file 1. [file 13071_2025_6943_MOESM1_ESM.tiff]

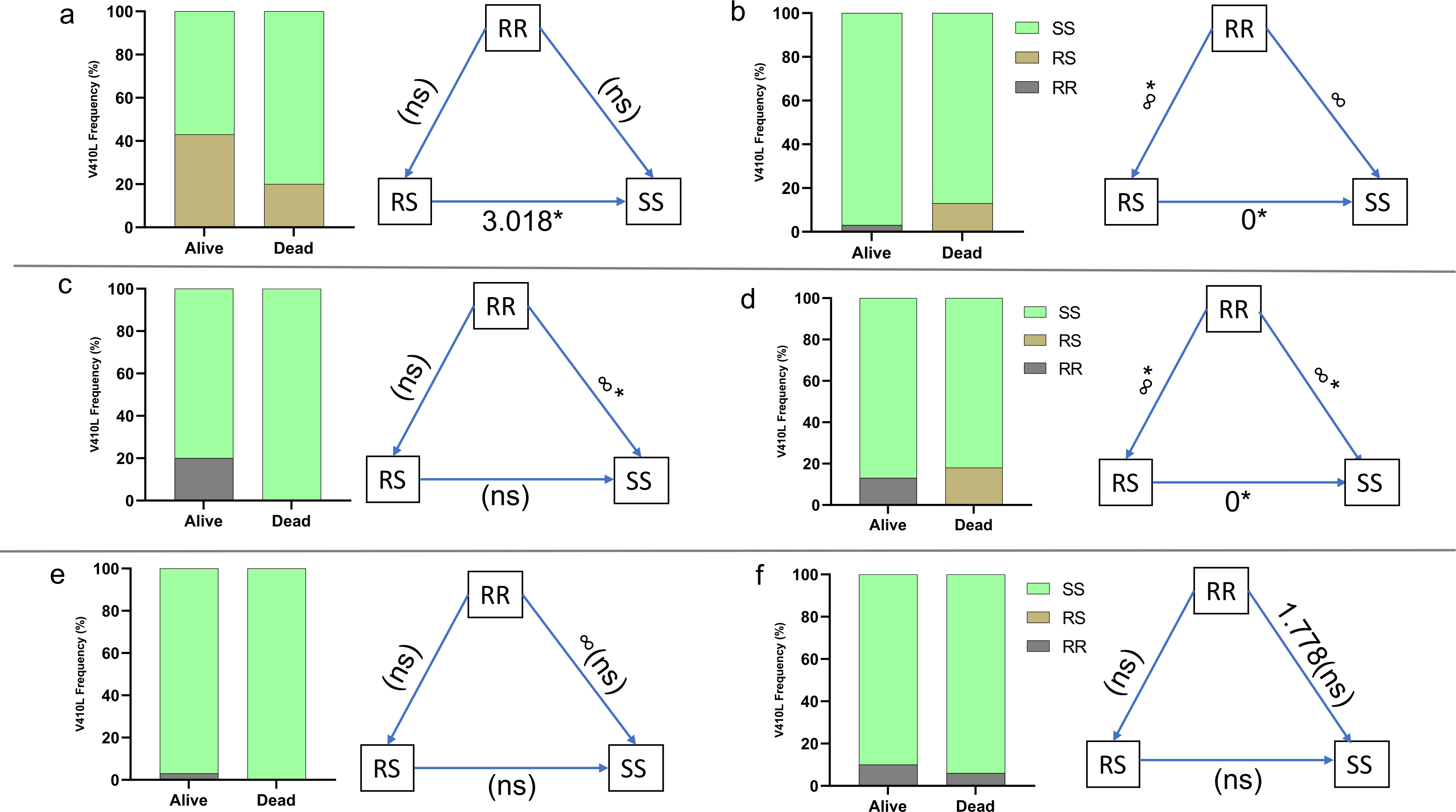

Supplement: Supplementary file 2 — Additional file 2. [file 13071_2025_6943_MOESM2_ESM.tiff]

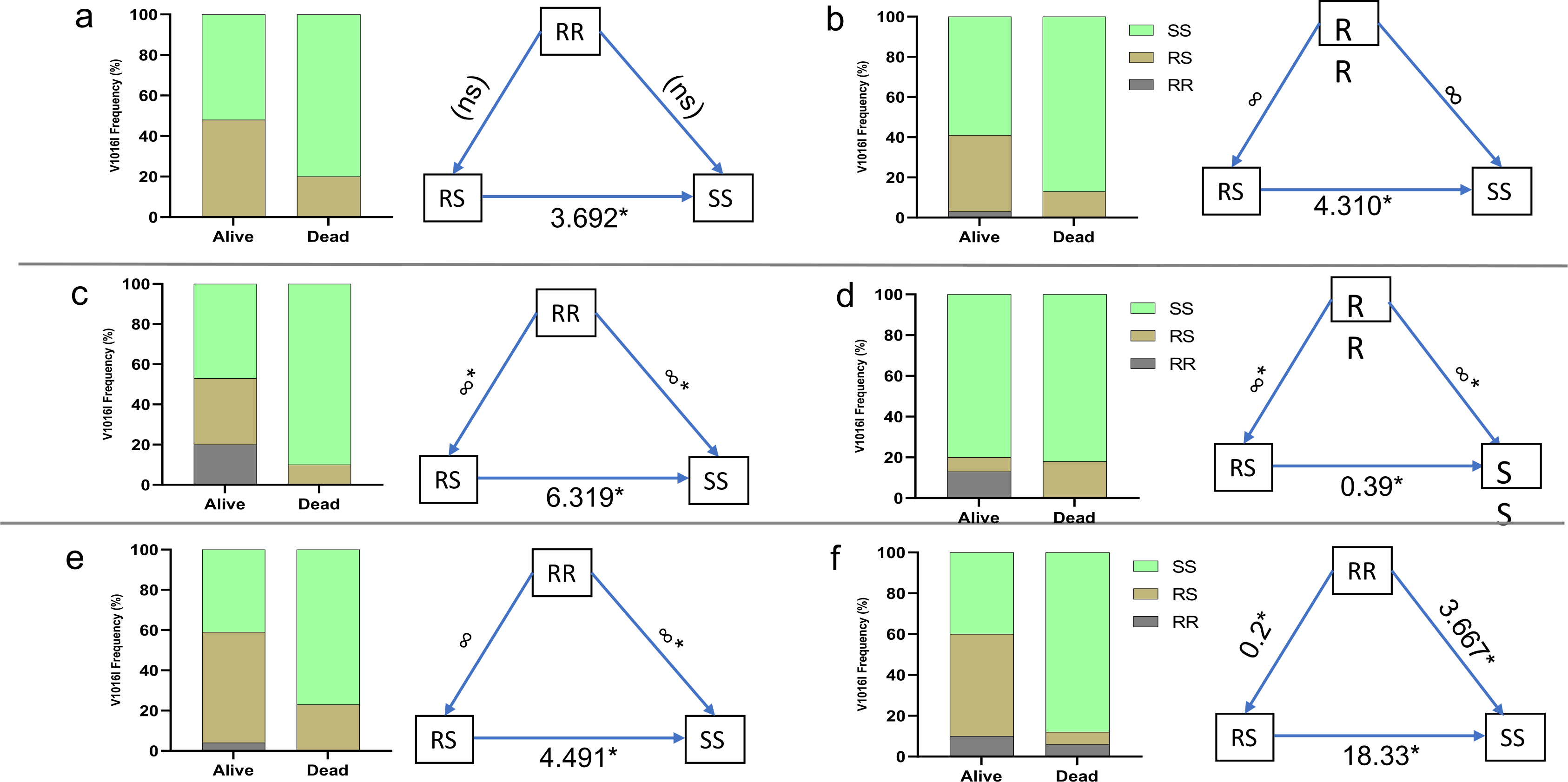

Supplement: Supplementary file 3 — Additional file 3. [file 13071_2025_6943_MOESM3_ESM.tiff]

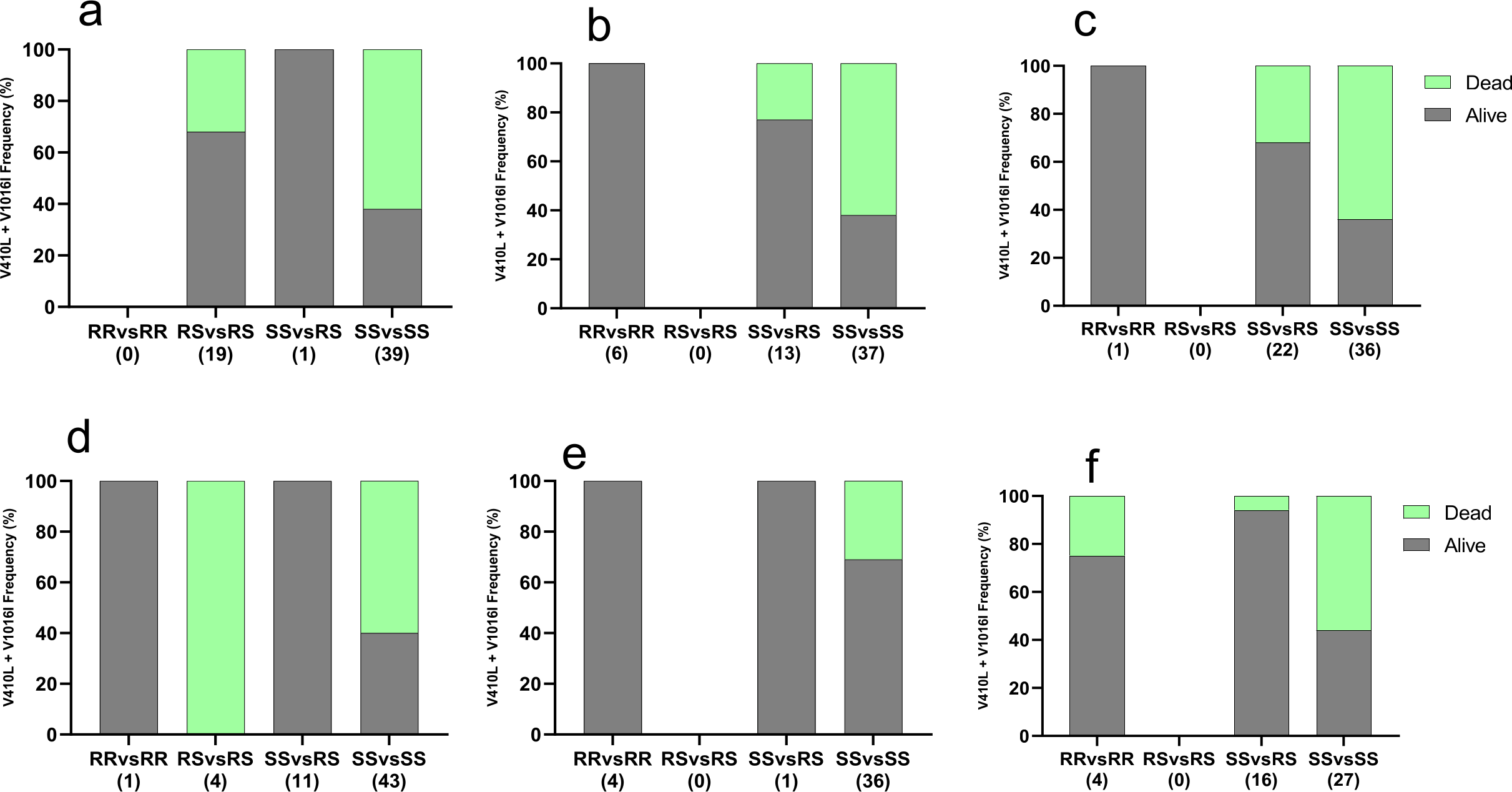

Supplement: Supplementary file 4 — Additional file 4. [file 13071_2025_6943_MOESM4_ESM.tiff]
