# Supplementary material for: Association of knockdown resistance mutations with pyrethroid resistance in Aedes aegypti, a major arbovirus vector in Cameroon
Source: Parasit Vectors. 2025 Jul 24;18:296. doi: 10.1186/s13071-025-06943-4 (PMC12291522; doi:10.1186/s13071-025-06943-4)
Supplement: Supplementary file 5 — Additional file 5. [file 13071_2025_6943_MOESM5_ESM.docx]

**Supplementary Table**

**Additional file 2 Table S1.** *F1534C* genotype and allele frequency distribution between dead and alive mosquitoes after exposure to pyrethroids;

n: number of sample; F: phenylalanine; C: cysteine; F/F: absence of the *F1534C* mutation; F/C: presence of the F1534C mutation with 2 alleles: one resistant, allele C and another susceptible F allele; C/C: presence of the *F1534C* mutation with the 2 resistant alleles; * : significant difference; ∞: Infinity; NA : not applicable

| **Samples** | **Insecticide** | **Phenotypes** | **n** | **Genotypes *F1534*C** | | | **Allelic frequencies** | | **Odds ratio** | **P value** |
| --- | --- | --- | --- | --- | --- | --- | --- | --- | --- | --- |
|  |  |  |  | **F/F** | **F/C** | **C/C** | **% F** | **% C** |  |  |
| Yaoundé | Deltamethrin | Alive | 30 | 0 | 1 | 29 | 1.66 | 98.32 | 14.64 | < 0.001* |
|  |  | Dead | 30 | 6 | 2 | 22 | 23.33 | 76.77 |  |  |
|  | Permethrin | Alive | 30 | 0 | 0 | 30 | 0 | 100 | ∞ | 0.0003* |
|  |  | Dead | 30 | 1 | 5 | 24 | 11.67 | 88.33 |  |  |
| Douala | Deltamethrin | Alive | 30 | 0 | 0 | 30 | 0 | 100 | NA | > 0.9999 |
|  |  | Dead | 28 | 0 | 0 | 28 | 0 | 100 |  |  |
|  | Permethrin | Alive | 30 | 0 | 0 | 30 | 0 | 100 | NA | > 0.9999 |
|  |  | Dead | 11 | 0 | 0 | 11 | 0 | 100 |  |  |
| Bertoua | Deltamethrin | Alive | 30 | 0 | 0 | 30 | 0 | 100 | ∞ | 0.2462 |
|  |  | Dead | 29 | 1 | 0 | 29 | 3.33 | 96.67 |  |  |
|  | Permethrin | Alive | 29 | 0 | 0 | 29 | 0 | 100 | NA | > 0.9999 |
|  |  | Dead | 18 | 0 | 0 | 18 | 0 | 100 |  |  |

**Additional file 3 Table S2:** *V410L* genotype and allele frequency distribution between dead and alive mosquitoes after exposure to pyrethroids;

n: number of sample; V: valine; L: leucine; VV: absence of the *V410L* mutation; VL: presence of the V410L mutation with 2 alleles: one resistant, allele L and another susceptible V allele; LL: presence of the *V410L* mutation with the 2 resistant alleles ; * : significant difference; ∞: Infinity;

| **Samples** | **Insecticide** | **Phenotypes** | **n** | **Genotypes *V410L*** | | | **Allelic frequencies** | | **Odds ratio** | **P value** |
| --- | --- | --- | --- | --- | --- | --- | --- | --- | --- | --- |
|  |  |  |  | **V/V** | **V/L** | **L/L** | **% V** | **% L** |  |  |
| Yaoundé | Deltamethrin | Alive | 30 | 17 | 13 | 0 | 78.33 | 21.67 | 2.538 | 0.0327* |
|  |  | Dead | 30 | 24 | 6 | 0 | 90 | 10 |  |  |
|  | Permethrin | Alive | 30 | 29 | 0 | 1 | 96.67 | 3.33 | 0.4109 | 0.3311 |
|  |  | Dead | 30 | 26 | 4 | 0 | 93.33 | 6.67 |  |  |
| Douala | Deltamethrin | Alive | 30 | 24 | 0 | 6 | 80 | 20 | ∞ | < 0.001* |
|  |  | Dead | 28 | 28 | 0 | 0 | 100 | 0 |  |  |
|  | Permethrin | Alive | 30 | 26 | 0 | 4 | 86.67 | 13.33 | 1.511 | 0.4986 |
|  |  | Dead | 11 | 9 | 2 | 0 | 90.9 | 9.1 |  |  |
| Bertoua | Deltamethrin | Alive | 30 | 29 | 0 | 1 | 96.67 | 3.33 | ∞ | 0.2462 |
|  |  | Dead | 30 | 30 | 0 | 0 | 100 | 0 |  |  |
|  | Permethrin | Alive | 30 | 27 | 0 | 3 | 90 | 10 | 1.741 | 0.4353 |
|  |  | Dead | 18 | 17 | 0 | 1 | 94.44 | 5.56 |  |  |

**Additional file 5 Table S3.** *V1016I* genotype and allele frequency distribution between dead and alive mosquitoes after exposure to pyrethroids;

n: number of sample; V: Valine; I: isoleucine; VV: absence of the *V1016I* mutation; VI: presence of the V1016I mutation with 2 alleles: one resistant, allele I and another susceptible V allele; II: presence of the *V1016I* mutation with the 2 resistant alleles; * : significant difference;

| **Samples** | **Insecticide** | **Phenotypes** | **n** | **Genotypes *V1016I*** | | | **Allelic frequencies** | | **Odds ratio** | **P value** |
| --- | --- | --- | --- | --- | --- | --- | --- | --- | --- | --- |
|  |  |  |  | **V/V** | **V/I** | **II** | **% V** | **% I** |  |  |
| **Yaoundé** | Deltamethrin | Alive | 29 | 15 | 14 | 0 | 75.86 | 24.14 | 2.842 | 0.0136* |
|  |  | Dead | 30 | 24 | 6 | 0 | 90 | 10 |  |  |
|  | Permethrin | Alive | 29 | 17 | 11 | 1 | 77.59 | 22.41 | 3.573 | 0.0043* |
|  |  | Dead | 30 | 26 | 4 | 0 | 93.33 | 6.67 |  |  |
| **Douala** | Deltamethrin | Alive | 30 | 14 | 10 | 6 | 63.33 | 36.67 | 9.201 | < 0.001* |
|  |  | Dead | 26 | 23 | 3 | 0 | 94.23 | 5.77 |  |  |
|  | Permethrin | Alive | 30 | 24 | 2 | 4 | 83.33 | 16.67 | 2.071 | 0.1400 |
|  |  | Dead | 11 | 9 | 2 | 0 | 90.9 | 9.1 |  |  |
| **Bertoua** | Deltamethrin | Alive | 29 | 12 | 16 | 1 | 68.97 | 31.03 | 3.295 | 0.0017* |
|  |  | Dead | 30 | 23 | 7 | 0 | 88.33 | 11.67 |  |  |
|  | Permethrin | Alive | 30 | 12 | 15 | 3 | 65 | 35 | 6.125 | < 0.001* |
|  |  | Dead | 17 | 15 | 1 | 1 | 91.18 | 8.82 |  |  |
